# Measurement and 3D-Visualization of Cell-Cycle Length Using Double Labelling with Two Thymidine Analogues Applied in Early Heart Development

**DOI:** 10.1371/journal.pone.0047719

**Published:** 2012-10-16

**Authors:** Bouke A. de Boer, Gert van den Berg, Alexandre T. Soufan, Piet A. J. de Boer, Jaco Hagoort, Maurice J. B. van den Hoff, Antoon F. M. Moorman, Jan M. Ruijter

**Affiliations:** Department of Anatomy, Embryology & Physiology, Academic Medical Center, Amsterdam, The Netherlands; Texas A&M University, United States of America

## Abstract

Organ development is a complex spatial process in which local differences in cell proliferation rate play a key role. Understanding this role requires the measurement of the length of the cell cycle at every position of the three-dimensional (3D) structure. This measurement can be accomplished by exposing the developing embryo to two different thymidine analogues for two different durations immediately followed by tissue fixation. This paper presents a method and a dedicated computer program to measure the resulting labelling indices and subsequently calculate and visualize local cell cycle lengths within the 3D morphological context of a developing organ. By applying this method to the developing heart, we show a large difference in cell cycle lengths between the early heart tube and the adjacent mesenchyme of the pericardial wall. Later in development, a local increase in cell size was found to be associated with a decrease in cell cycle length in the region where the chamber myocardium starts to develop. The combined application of halogenated-thymidine double exposure and image processing enables the automated study of local cell cycle parameters in single specimens in a full 3D context. It can be applied in a wide range of research fields ranging from embryonic development to tissue regeneration and cancer research.

## Introduction

**Figure 1:**
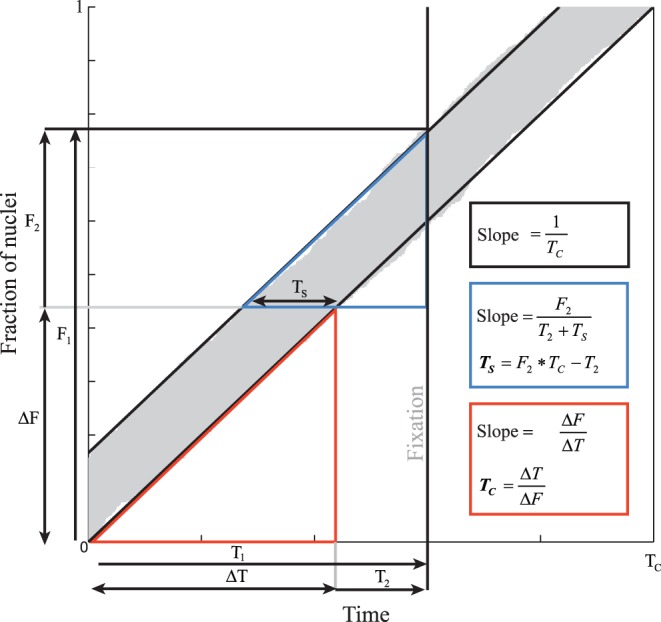
Graph to illustrate the differential exposure time theorem. The graph is based on a simulation of 500 nuclei with a fixed S-phase length and cell cycle length randomly entering S-phase. The S-phases are then sorted and plotted in gray. The Y-axis has a length of 1 (all cells) and the X-axis has a length of one cell cycle (T_C_). The slope of the line formed by the end points of the S-phases is therefore by definition 1/T_C_. Thymidine analogues are incorporated into the nucleus during the S-phase (between the black lines). The fraction of labelled nuclei is thus determined by the length of the S-phase plus the time of exposure to the thymidine analogue divided by the total length of the cell cycle [7]. When two different exposure times are applied, either by two different thymidine analogues or in two different animals, the difference in fraction of labelled nuclei (ΔF) is linearly related to the difference in exposure time (ΔT) (red triangle). Because the slope of this relation is equal to 1/T_C_, the cell cycle length can be calculated (red box). Similarly, the length of the S-phase can be determined from the slope in the blue triangle and the calculated T_C_ (blue box).

To understand growth and morphogenesis during embryonic development it is essential to know local differences in morphogenetic parameters like cell size, cell cycle length and growth fraction. Labelling of proliferating cells in the embryo has been used to demonstrate local differences and stage-dependent changes in fraction of labelled cells (F), or labelling index (LI), in the developing heart [1]–[5]}. These labelling indices have been interpreted to reflect proliferation rate. However, when the labelling index is the result of the staining of an event that only occurs during a specific phase of the cell cycle, the index is merely proportional to the fraction of cells in that phase. This not only holds for phosphorylated *histone* H3 staining to identify cells in M-phase [3] or counting of mitotic figures [1], but also for modern molecular approaches [6]. The obtained results do not allow for the calculation of cell cycle length or proliferation rate because multiple time-based parameters are unknown. E.g. the index resulting from counting the number of nuclei that are labelled with BrdU, and thus were exposed to the label during the S-phase, is not only dependent on the duration of the exposure, but also on the lengths of the S-phase and the cell cycle [7]. Consequently, differences in exposure time hamper the comparison of such labelling indices between experiments. On the other hand, the use of different exposure times enables the calculation of cell cycle length and S-phase length [4], [7], [8]. In these studies, several embryos were exposed to a single radioactive or halogen-conjugated thymidine analogue for different lengths of time before sacrifice. When the dividing cells can be assumed to be in a random phase of the cell cycle, the cell cycle length is constant and the population does not increase in size during the exposure time [8], the relation between labelling index, exposure time (T_exp_), cell cycle length (T_C_) and S-phase length (T_S_) can be described by a linear equation: LI = (T_S_+T_exp_)/T_C_ [7]. As shown in Figure 1, the slope and intercept of this relation can be used to calculate the cell cycle length and S-phase duration, respectively. However, previous application of this procedure only results in average estimates of these parameters within a pre-defined region-of-interest [4], [8]. When using a single thymidine analogue for estimation of local cell cycle lengths in a developing organ or embryo it is required to apply a 3D registration of the different specimens [7]. The latter is close to impossible in complex and fast developing organs such as the heart. However, by exposing a single embryo to two distinct labels and different exposure times [9], [10], the differential exposure time theorem (Fig. 1) for the estimation of cell cycle length can be applied to a single organ or specimen This approach has been applied in a study of the mouse limb bud in which local manual measurements of the cell cycle length were extrapolated to obtain a 3D pattern of the proliferation gradient [11].

In this paper we report the double exposure to halogenated thymidines, the automated full three-dimensional (3D) measurement and the visualization of local cell-cycle lengths (in vivo, single embryo’s) using 3D reconstructions from sections. We describe the differential nuclear incorporation of two different halogenated thymidine analogues (e.g. BdrU, IdU, CldU or EdU) in one tissue [9], [10], [12]. The program used for the detection of labelled nuclei, the spatial determination of labelling fractions and the calculation of local cell cycle length is presented and made available. We apply this technique to embryonic heart development, showing that the cells in the early heart tube have a long cycle length and that chamber development is marked by a local increase in proliferation rate with cell cycle lengths as short as 8 hours.

## Materials and Methods

Chicken embryos were obtained by timed incubation of fertilized eggs (Drost Loosdrecht B.V.), and staged according to Hamburger and Hamilton [13]. To prepare equimolar (32 mM)solutions of CldU and IdU were prepared either 8.6 mg CldU (Sigma, nr. C6891) was dissolved per ml of sterile physiological salt solution or 23 mg of IdU (Sigma, nr. I7125) was dissolved in 1 ml of 0.1 N NaOH and then neutralized with 0.8 ml of 1.5% NaCl and 0.2 ml of 0.3 N HCl.

To expose the embryos to the two thymidine analogues, 100 µl of IdU solution was injected into the yolk sack of incubated chicken eggs which were then placed back into the incubator. After 3 hours, an injection with 100 µl of CldU solution followed. After another hour, the embryos were isolated and washed in chicken physiological salt solution (0.719% NaCl).

The embryos were then fixed in Methanol-Acetone-Water (40/40/20 vol/vol/vol), followed by dehydration, embedding in paraplast and sectioning at 7 µm. Antigens were retrieved by 5 min of pressure cooking in antigen unmasking solution (Vector H3300). Each section was exposed overnight to a mixture of anti-IdU (mouse-monoclonal anti-BrdU; BD, 347580), anti-CldU (rat-monoclonal anti-BrdU; Serotec, OBT0030CX) and anti-cTnI (rabbit polyclonal; HyTest, 4T21/2) followed by incubation for at least 2 hrs with a mixture of the fluorescent antibodies, goat-anti-mouse-Alexa 680, goat-anti-rat-Alexa 568, goat-anti-rabbit-Alexa 405 (Invitrogen), and Sytox green 488 (Invitrogen). Image acquisition was performed with a fluorescent microscope using a 4 channel setup (Leica DM6000, Chromaphor).

Morphological 3D reconstructions were made using Amira (Visage Imaging) as previously described [14]. The myocardium was segmented based on cTnI expression; the dorsal mesoderm flanking the heart was manually segmented based on morphological landmarks. Both structures were separately used as masks for cell counting. Cell counting was performed in cubic sub-volumes (referred to as boxels) to ensure that enough nuclei were counted to reach reliable labelling indices [15]. The number of cells per boxel was used to calculate a local average cell size [3]. Cell counting and cell cycle length computation were done using a custom written Matlab (Version 2009a, The Mathworks) program (see Results; available under GPL licence: http://downloads.hfrc.nl).

The resulting quantitative information was imported into Amira and projected onto the morphological reconstruction of the embryonic heart, which is based on cTnI staining, and manual segmented surrounding tissue. These reconstructions were incorporated into an interactive 3D pdf file [16].

### Cluster Analysis

A cluster analysis was performed on boxels using average cell size and cycle length per boxel as variables. K-means clustering was applied on standardized data using squared Euclidean distance as distance measure. The known x, y and z positions of each boxel were used to plot cluster membership onto the 3D reconstruction.

## Results

### Differential Exposure Time Theorem

The principle of the differential exposure time theorem is illustrated in Figure 1. The main assumptions are similar to those of the exposure of different specimens: the dividing cells are assumed to be in a random phase of the cell cycle, cell cycle length is constant and the population does not increase in size during the exposure time [8]. Under these assumptions, all cells pass once through the cell cycle when 1 T_C_ has passed and thus the slope of the line that connects the end of the (sorted) S-phases is equal to 1/T_C_ (Fig. 1). This slope is also equal to the difference in labelling fractions (ΔF) divided by the difference in exposure times (ΔT) (red triangle in Fig. 1) which allows the calculation of T_C_ as ΔT/ΔF (see also the mathematical appendix).

### Labelling Efficiency and Cross Reactivity

**Figure 2:**
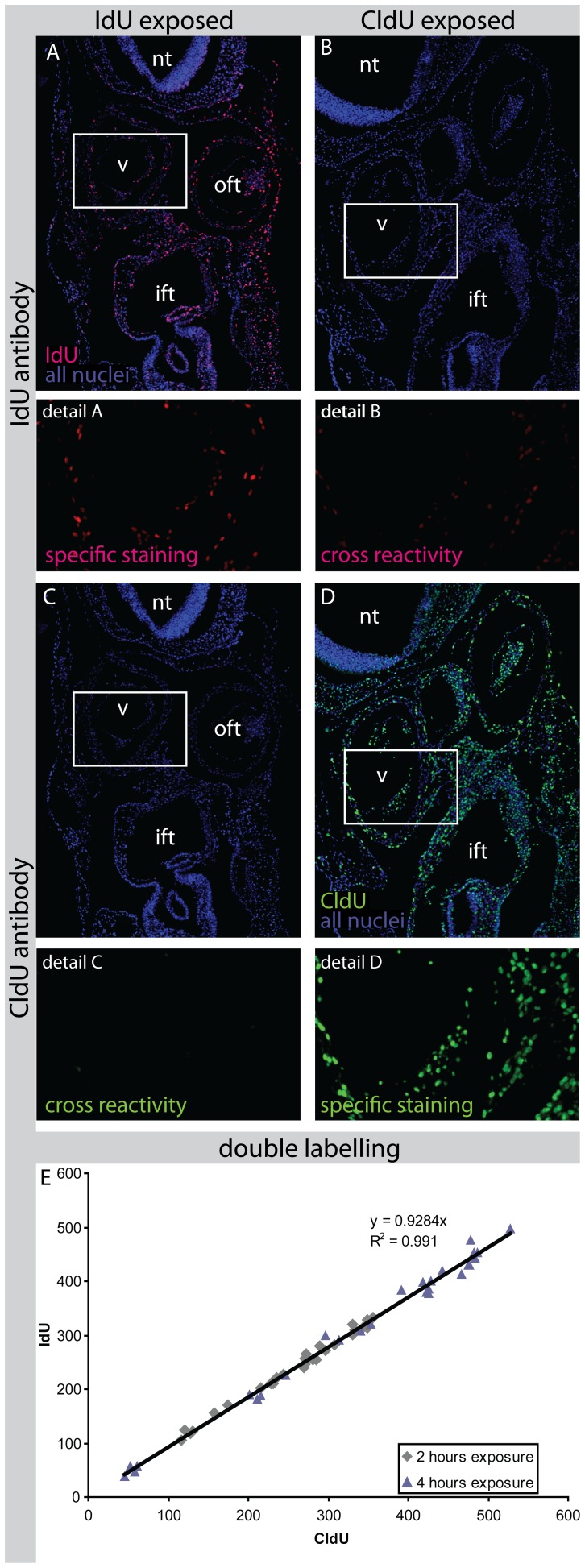
Cross-reactivity and validation of antibody staining. Section of an embryo exposed to IdU shows specific staining using an antibody against IdU (Panel A). When an antibody against CldU is used there is no aspecific staining visible (Panel C). When an embryo is exposed to CldU and an antibody agains IdU is used some cross reactivity is observed (Panel B). Panel D shows specific staining for CldU. Abbreviations: ift: Inflow Tract; nt: Neural Tubel; oft: Outflow Tract; v: Ventricle. Panel E shows the relation between the number of nuclei labelled for IdU and for CldU at equal exposure times. Each point represents a section. There was no significant difference between 2 and 4 hours of exposure time. The linear relation shows a high correlation coefficient (R^2^ = 0.991) and detection of 7.2% less IdU than CldU positive nuclei.

A requirement for quantitative analysis of proliferation is the equimolar administration of IdU and CldU [12]. Because the antibody against IdU showed a cross reaction to CldU (Fig. 2B) and the antibody against CldU did not cross react with IdU (Fig. 2C) we chose to expose the embryos for 4 hours to IdU, the last hour combined with exposure to CldU. As a result, all CldU labelled nuclei were also IdU-labelled which made cross reactivity between the IdU antibody and CldU irrelevant.

Double labelling experiments with equal exposure time to IdU and CldU demonstrated that the number of detected IdU-positive nuclei was 0.928 (95% CI: 0.919–0.938) times the number of CldU-positive nuclei (Fig. 2E). Since this was a constant difference, independent of exposure time, a 7.2% compensation was implemented in the calculation of the IdU-positive fraction.

### Proliferation Toolbox

A program was developed to automate the steps needed for measurement and visualisation of the cell cycle lengths (available for download from hfrc.nl). A two step procedure needs to be followed. At first the single nuclei need to be detected and their labelling with the thymidine analogues needs to be determined. In the second step the quantitative measures, labelling indices, cell size and cell cycle length, are computed for each sub-volume of the dataset. The quantitative data is projected onto the original labelled sections or, alternatively, onto 3D surface files using an external program. Input files for the program are 4 sets of aligned images. One set in which different materials (or labels) are assigned, one set with a nuclear staining and a set for each thymidine analogue.

### Detection of Nuclei

**Figure 3:**
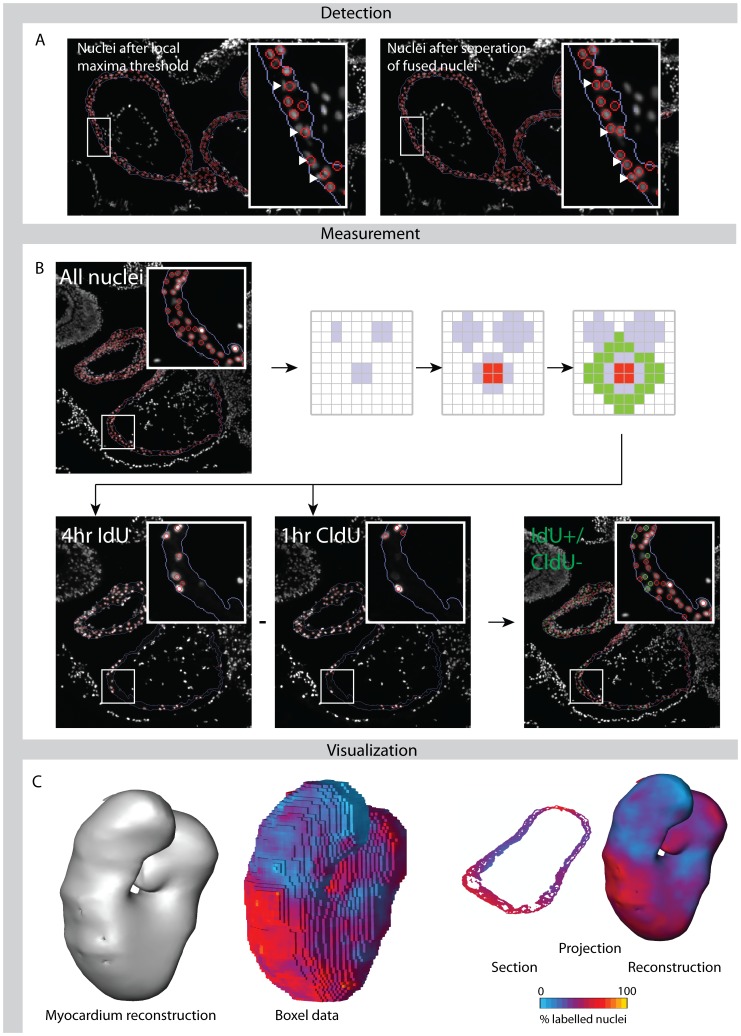
Image analysis and visualisation. Panel A. Using a Sytox green staining, all nuclei are first detected based on a local-maxima threshold. All detected objects that were at least twice as large as the median object size were processed to separate these fused nuclei (inserts). Panel B shows a schematic overview of the image processing steps involved in the recognition of IdU- and CldU-positive nuclei. After the detection of the Sytox green stained nuclei, each nucleus is individually processed. A zone is selected around all nuclei which will be excluded in the following measurement (gray zone). For each nucleus within the region of interest (myocardium), the algorithm measures the signal in (red area) and around (green area) the nucleus in the IdU and CldU channels. The measurement of the local background excludes the locations at which other nuclei were detected (gray zone). When the signal in the nucleus is at least a standard deviation above the background, the nucleus is classified as positively labelled. The program generates control images both for the nuclei detection as well as for which nuclei are positive for the proliferation markers. The difference between the two proliferation markers is used to determine ΔF (number of green nuclei divided by total number of nuclei). Panel C shows how the quantitative information can be projected onto a reconstruction or onto the original section. Each unit in the boxel representation has a volume of approximately 21^3^ µm^3^, and is the central boxel of the sampling volume of approximately 105^3^ µm^3^ that is required for reliable measurement of the labelling indices [15].

The nuclear staining with Sytox-green enabled the detection of individual nuclei (Fig. 3A). Noise was removed by application of a 3×3 pixels Wiener filter, as implemented in Matlab. The noise variance used in the filter was the average of all locally estimated variance values which were computed using the 3×3 box. Background staining was removed by subtracting a moving average image; the size of the rectangular shaped filter used was in our case 10×10 pixels, which is a user setting and is ideally of the same size as a nucleus. The latter step is fundamental as it makes the detection of nuclei independent from the gradient of background staining, which is often present in biological samples. A threshold was set on the local maxima image, resulting in a binary image in which the majority of the objects will be single nuclei. However, nuclei in close proximity to each other might have fused to larger binary objects. To resolve these objects, the median object area was computed. Objects larger then twice the median area were considered to be possibly multiple nuclei and were separated using, in short, grey value erosion, dilation and reconstruction [17]. Figure 3A shows that this method effectively separates these fused nuclei. Labelled control images enabled the validation of the image processing steps (Fig. 3A).

After correct localization of the nuclei it was determined whether the nuclei are positively stained for IdU, CldU or both (Fig. 3B). We considered a nucleus to be positive when the mean intensity of the IdU or CldU signal in this nucleus was at least the mean standard deviation (sd) of all background regions higher than its local background; thus for every detected nucleus the difference in mean intensity between the nucleus and its local background was standardized with respect to the overall background. This computationally intensive method was necessary because of the speckled nuclear staining pattern of CldU and in particular IdU (Fig. 3B).

For each experiment, the detection limit was re-calibrated, compensating for experiment dependent variation. This calibration was done by setting the detection thresholds for IdU on 1 sd and for CldU to a value where the fraction IdU-labelled nuclei within the CldU-labelled population approached 0.928. Changing the detection threshold to higher values has little impact on the computed cell cycle lengths (Figure S1). Nuclei and nuclei positive for IdU or CldU were reduced to the center pixel of the nuclei. Control images were generated to enable the user to check the assignment of positive and negative nuclei (Fig. 3B.).

### Quantitative Measurements and Visualization

To calculate cell cycle length in 3D volumes, aligned stacks of images were required [14]. Calculations were done in cubic sample volumes of 105^3^ µm^3^, which were previously shown to contain enough nuclei to determine reliable labelling indices [15]. To preserve spatial resolution, results were then assigned to the central 21^3^ µm^3^ volume which we refer to as boxels. The sample volume is systematically moved 21 µm in X, Y and Z directions to obtain the required data for each boxel containing tissue. Cell cycle lengths were determined using the formulas in Figure 1 (see also the mathematical appendix). This measurement procedure results in a 3D representation of the labelling indices covering the full tissue space (Fig. 3C, boxel data). The number of observed nuclei per tissue area depends on the number of nuclei per volume, the diameter of these nuclei and the section thickness. To enable the computation of the average cell size per sample volume, the observed number of nuclei per tissue area was converted into the number of nuclei per volume according to Abercrombie’s equation [18]. To calculate the average cell size within a boxel, the tissue volume within the boxel was divided by the number of nuclei per volume determined for this boxel.

Two ways of visualization are possible (Fig. 3C). One option, which is part of the program, is to project the quantitative information onto the original segmented sections which allows direct comparison of calculated cell cycle lengths with the acquired fluorescence images. However, the more intuitive way of visualisation is the 3D visualisation in which the quantitative information is projected onto the morphological reconstruction.

### Application in Early Cardiac Development

**Figure 4:**
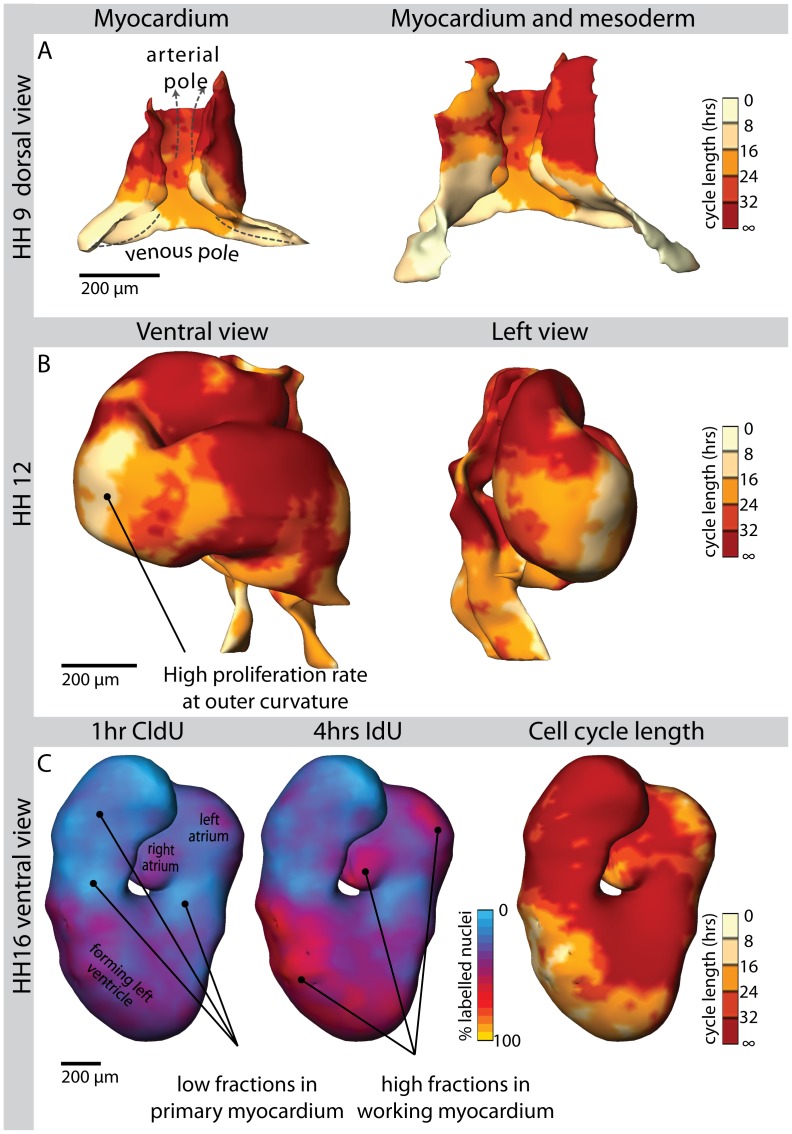
Application in heart development. 3D visualisation of cell cycle length in the heart at stages HH9 (Panel A), HH12 (Panel B) and HH16 (Panel C) of chicken embryonic development. The pointer in panel B indicates the region with a high proliferation rate at the site of early ventricle formation. Panel C shows the quantitative reconstructions of the individual labelling indices for CldU and IdU, on which the cycle lengths are based. The pointers in the CldU reconstruction indicate areas in which a low fraction of cells is positive in both CldU and IdU reconstructions, resulting in a low labelling difference and thus a long cell cycle length. The pointers in the IdU reconstruction show large differences in IdU and CldU labelling indices, indicating short cell cycle lengths. Note the heterogeneity in cell cycle lengths in different parts of the heart at every stage. Interactive versions of the 3D-reconstructions can be found in Interactive 3D-pdf S1.

The heart tube forms from the precursors from the first heart field which is located in the lateral plate mesoderm. From the heart fields a myocardial heart tube formed around an endocardial lumen. This tube consists out of primary myocardium which does not express chamber specific genes. The early primary myocardium at stage HH9 (according to Hamburger and Hamilton [13]) showed low proliferation rates with cell cycle lengths higher than 32 hours. In contrast, the neighbouring caudal part of the dorsal mesoderm displayed a much higher proliferation rate with cell cycle lengths as short as 8 hours (Fig. 4A and Interactive 3D-pdf S1). At HH12, less than one day later in development, the S-shaped heart showed a localized band with a high proliferation rate at the outer curvature (Fig. 4B and Interactive 3D-pdf S1); the position of this band corresponds to the known site of onset of cardiac chamber formation. The primary myocardium, which is still present at the inflow, outflow and inner curvature, showed large areas with cell cycle lengths of over 32 hours. At the caudal part of the dorsal mesoderm, relatively high proliferation rates persisted. At stage HH16 (Fig. 4C and Interactive 3D-pdf S1), high proliferation rates were not only found in the embryonic ventricle, but also in the newly forming atria. At this stage, the primary myocardium still retained its low proliferation rate.

**Figure 5:**
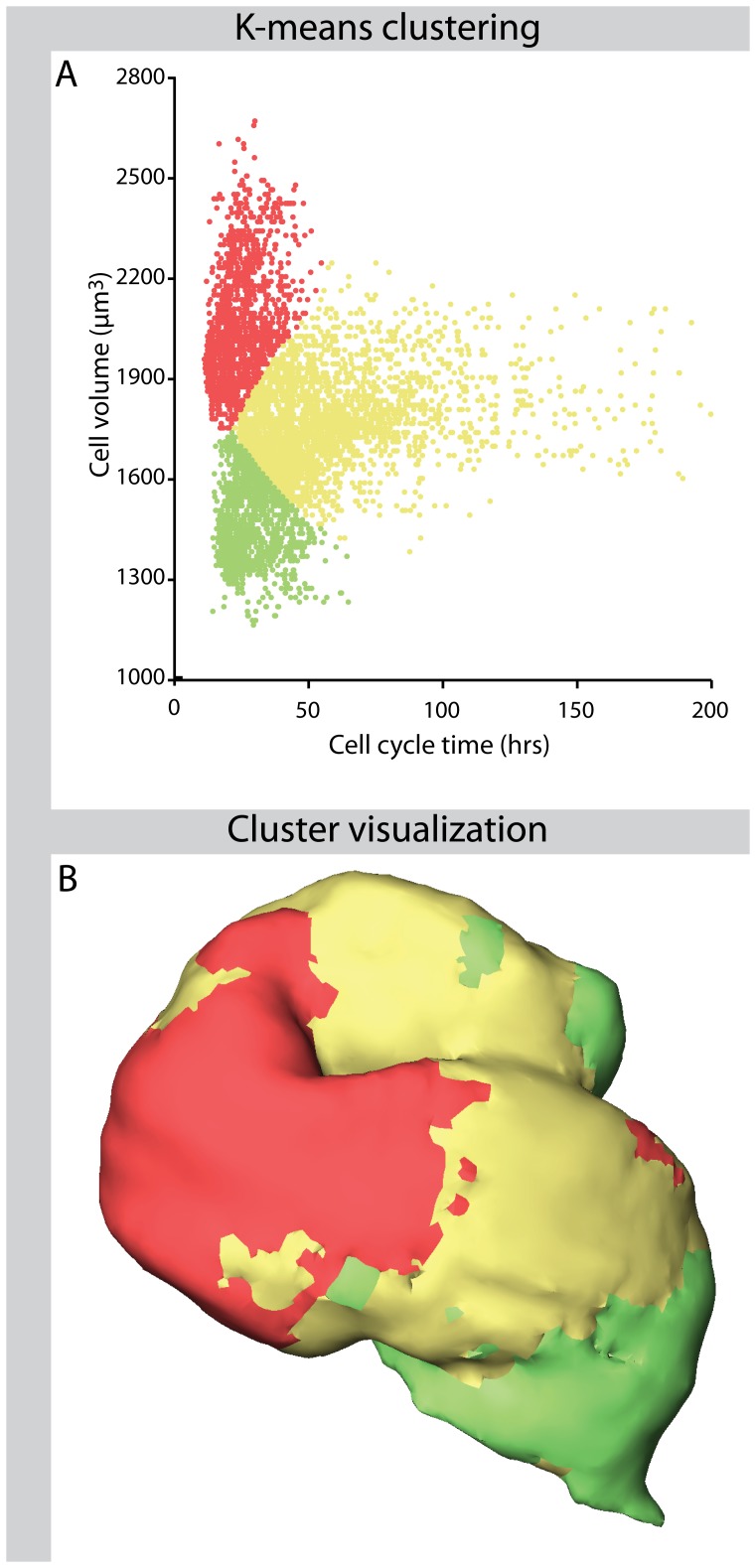
Cell size - cell cycle length clustering and visualisation. Panel A shows a k-means cluster analysis of boxels based on their cell volume and their cell cycle length. Panel B shows the result of plotting cluster membership of each boxel into the HH12 myocardium reconstruction. Although spatial information was not used in the cluster procedure, the resulting clusters show clear spatial continuity.

To determine whether a relation between cell size an cell cycle length exists a cluster analysis was performed on the boxels based on the average cell size [3] and cycle lengths in each boxel (Fig. 5A). In the HH12 myocardium, this analysis revealed three clusters of boxels with small fast-cycling cells, larger slow-cycling cells and large fast-cycling cells, respectively. Spatial mapping of the resulting clusters of boxels showed that they form spatially separated populations (Fig. 5B). At the inflow and outflow regions of the heart, the small rapidly proliferating cells were found, whereas the large and fast cycling cells were located at the developing ventricle. The medium sized slowly proliferating cells were found in the remaining primary myocardium.

## Discussion

### Methodological Considerations

Traditionally, cell cycle parameters were determined by pulse-chase experiments or differential exposure times using (radioactive) thymidine analogues in vivo or in vitro [19]–[21]. Another approach to determine cell cycle lengths is to use different exposure times to a single thymidine analogue, either by single [4], [7], [22] or by repetitive injections [8], [23]. For the latter approaches it is necessary to measure labelling indices in different specimens which was shown to lead to standard errors in T_C_ which were 5 to 10 times larger than when double labelling in one specimen was performed [9]. The smaller variation of the double labelling approach can be explained by the fact that the double labelling is not affected by developmental variation between specimens. Moreover, the random sampling error differentially affects independently counted nuclei in different specimens; in double labelling the fractions of CldU and IdU positive cells are based on the same total number of nuclei, and thus share the same sampling error which propagates only once in ΔF. Therefore, the analysis of the labelling index precision of single label experiments performed by Soufan et al. [15] also apply to the current cell cycle length computation.

The average cell cycle length in defined compartments was determined from the labelling indices measured in different specimens at at least two different time points [7]. The most important innovation of the current study is the application of this differential exposure time theorem to determine cell cycle lengths at each location in a single specimen preserving the 3D morphology. Therefore it is no longer necessary to subdivide a specimen into compartments [4], [8], [23], which might introduce a bias based on assumed compartment borders, or to align different specimens [7] by which biological variation between specimens is a source of error. This new approach was made possible by the independent detection of two different non-radioactive thymidine analogues [9], [12], [20]. The automated assignment of labelling indices, enabled by the fluorescent labelling of the incorporated thymidine analogues [20], obviated manual image processing [10], [11]. The size of the sampling volumes was chosen to yield the number of cells required for reliable determination of a labelling fraction [15] and determines the spatial resolution of the resulting quantitative reconstructions. The numerical precision of the measurement is only determined by the total number of cells, not by the labelled cells [15].

When measuring cell cycle lengths, an important factor to keep in mind is the growth fraction, which is the fraction of actively cycling cells. The mathematical appendix shows that disregarding the growth fraction results in the population doubling time instead of the cell cycle length. To quantify and model growth [11], the numerical impact of cell cycle length combined with the growth fraction is equal to the impact of population doubling time. However, when studying the regulation of growth, one should consider that the fraction of cycling cells and the actual length of the cell cycle are different biological parameters. Measurement of local cell cycle lengths therefore requires that, together with the halogenated-thymidine labelling indices, the growth fraction in each sub-volume is determined using a cell cycle marker like Ki67 [24].

The observed cell cycle length is not biased when the thymidine analogues are not instantly incorporated into cycling cells as we show in the mathematical appendix. In an experiment with different exposure times, we observed that, from 15 minutes BrdU exposure onwards, the resulting BrdU incorporation follows a linear relation (data not shown). This also showed that a 15 minutes exposure time is enough to reach a reliably detectable level of incorporation; in cell culture, IdU and CldU uptake required only 2 min [20].

The main assumptions of this double labelling method are that the dividing cells are in a random phase of the cell cycle, the cell cycle length is constant and the population does not increase in size during the exposure time [7], [8], [23].

The assumption that all cells are at a random phase of the cell cycle and the cell cycle length is constant may, however, not hold at every location in the developing heart. We know for instance that cells move from the highly proliferative growth centre in the flanking mesoderm into the low proliferative heart tube [4]. At the borders between the growth centre and the heart tube, the cell cycle length increases. In those border zones, the calculated cell cycle lengths may be affected by this change in cell cycle length during the exposure time. Therefore, in developmental studies, results should always be interpreted in the context of known morphogenetic processes. However, with the measurement in boxels, which contain relatively small volumes, only boxels in close proximity to those border zones will be affected.

To avoid the complication that the population of labelled cells increases as a result of division of labelled cells the exposure time should be shorter than the length of the G2 plus M-phase. When this requirement is not met, cycle lengths will be underestimated (see mathematical appendix). Finally, the method assumes that the measurements are done in a closed population of cells. As long as cells move as a coherent sheet, the local measurement is not affected by this movement. The implementation of measurements in sub-volumes (boxels) of tissue [7], [15] circumvents this assumption in most cases because within each sub-volume the cells will usually share similar history and properties. However, migrating cells, such as neural crest cells, cross into other tissues, and may change the population during the thymidine exposure. In most cases this problem can be tackled using specific markers to define the tissue-of-interest. In our current approach, we used a cTnI staining to select myocardial tissue and only counted nuclei in cTnI expressing cells. This approach can be used to study proliferative behaviour of any sub-population of cells that plays a role in organ development.

### Biological Application

In the caudal part of the dorsal mesoderm flanking the heart we found high proliferation rates. This is in agreement with previously described high labelling indices in the developing chicken embryo [4] and the high percentage of mitotic figures in this region [1]. In this part of the mesoderm we found cycle lengths that are similar to the previously described 7–7.5 hours for rat mesoderm [25].

The short cell cycle lengths found in the developing ventricle are also in agreement with the high labelling indices described previously [3], [4]. This embryonic ventricle shows relatively high proliferation rates, although we found slightly longer cycle lengths than the average of 8.5 hours found by using a cumulative BrdU labelling in different animals [4]. The latter low estimate is likely to be the result of exposure times to BrdU (up to 6 hours) being probably longer than the summed lengths of the G2 and S-phase.

Soufan and co-workers found a relation between cell size and BrdU labelling index showing small cells with high labelling indices at the inflow and ouflow region, medium sized cells with low labelling indices in the primary myocardium and large cells with high labelling indices at the developing ventricle [3]. We found similar populations after clustering of cell cycle lengths and cell size. The small rapidly cycling cells are just added to the heart [26] which suggests that the cell cycle length of those cells was regulated in the heart-forming undifferentiated mesoderm. The regions with slowly cycling cells are adjacent to these small rapidly cycling cells whereas the large rapidly cycling cells are located in the middle of the heart tube, which consists out of the oldest myocardium. This pattern indicates that the small cells that are added to the heart first have to grow before proliferation is reinitiated in the forming ventricle.

### Concluding Remarks

Although we were not yet able to distinguish the actual cell cycle length from population doubling time, the current application shows clear heterogeneity in population doubling times in different parts of the developing heart. Our method enables the automated study of local cell cycle parameters in single specimens in a genuinely 3D context. It can be applied in a wide range of research fields ranging from embryonic development to tissue regeneration and cancer research.

## Supporting Information

Figure S1 **Cell cycle lengths in a HH12 chicken heart based on 3 different threshold settings**. The threshold for IdU-positivity was set to 1 sd (A), 2 sd (B) and 3 sd (C). As described in the Methods, the threshold for CldU was calibrated based on the percentage of double labelling and was 1.4, 3.6 and 6.2 sd, respectively. Changing the threshold setting had little impact on the proliferation pattern or observed cell cycle lengths.
(TIF)

Interactive 3D-pdf S1 **Interactive cell cycle length reconstructions.** 3D reconstructions of cell cycle length of embryonic chicken heart of stages HH9, HH12 and HH16 in 3D-pdf format. This format allows the user to interactively explore the 3D structure of the developing heart.
(PDF)

Appendix S1 **Mathematics of labelling indices.** This appendix describes the derivation of the equations used to calculate the cell cycle length and S-phase length from the labelling indices that can be observed after differential exposure of dividing cells to thymidine analogues.
(PDF)
