# Supplementary figures and images for: Measurement and 3D-Visualization of Cell-Cycle Length Using Double Labelling with Two Thymidine Analogues Applied in Early Heart Development
Source: PLoS One. 2012 Oct 16;7(10):e47719. doi: 10.1371/journal.pone.0047719 (PMC3473012; doi:10.1371/journal.pone.0047719)

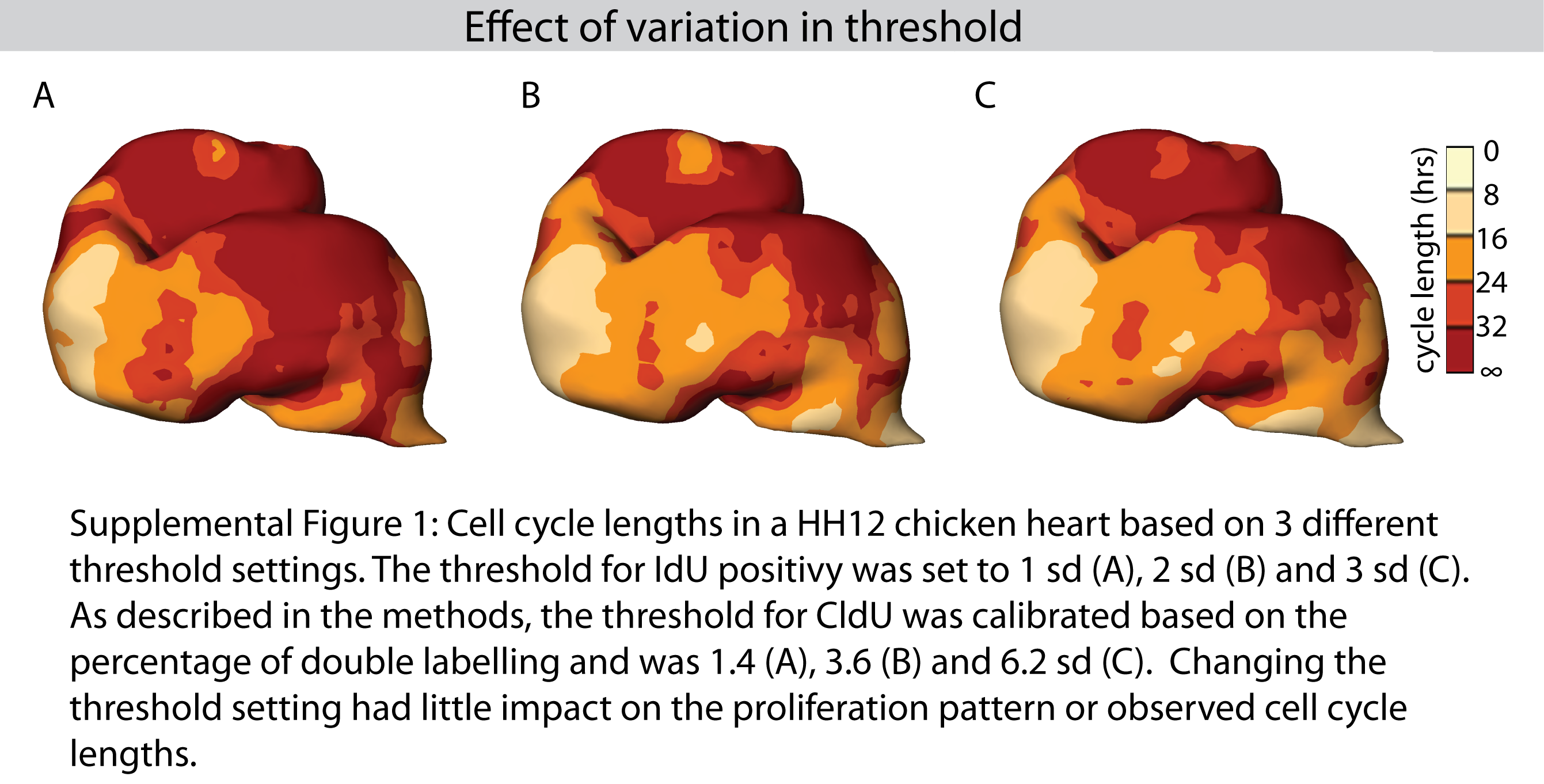

Supplement: Figure S1 — Cell cycle lengths in a HH12 chicken heart based on 3 different threshold settings. The threshold for IdU-positivity was set to 1 sd (A), 2 sd (B) and 3 sd (C). As described in the Methods, the threshold for CldU was calibrated based on the percentage of double labelling and was 1.4, 3.6 and 6.2 sd, respectively. Changing the threshold setting had little impact on the proliferation pattern or observed cell cycle lengths. (TIF) [file pone.0047719.s001.tif]
