# Supplementary material for: Measurement and 3D-Visualization of Cell-Cycle Length Using Double Labelling with Two Thymidine Analogues Applied in Early Heart Development
Source: PLoS One. 2012 Oct 16;7(10):e47719. doi: 10.1371/journal.pone.0047719 (PMC3473012; doi:10.1371/journal.pone.0047719)
Supplement: Interactive 3D-pdf S1 — Interactive cell cycle length reconstructions. 3D reconstructions of cell cycle length of embryonic chicken heart of stages HH9, HH12 and HH16 in 3D-pdf format. This format allows the user to interactively explore the 3D structure of the developing heart. (PDF) [file pone.0047719.s002.pdf]

How to use this interactive 3D pdf

views

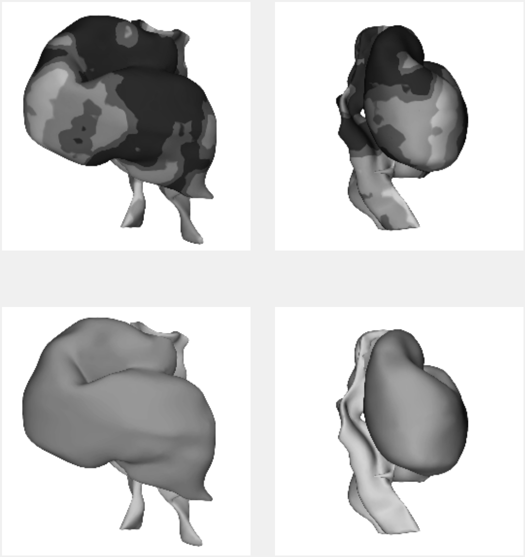

structures

myocardium

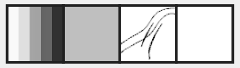

mesoderm

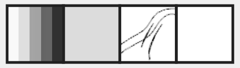

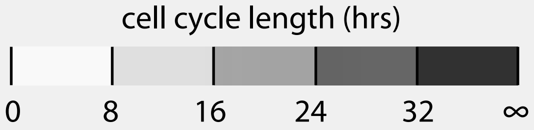

http://3d.hfrc.nl

To interact with the reconstruction on the next page

Rotate: Hold left mouse-button and move mouse.

Zoom: Hold right mouse-button and move mouse up or down.

Translate: Hold left and right mouse-buttons and move mouse.

Selection of preset views

The views panel contains several preset views.
Click to select such a view.

Selection of structures

The structures panel contains buttons for each structure to hide or show this structure with overlay data, solid or transparent.

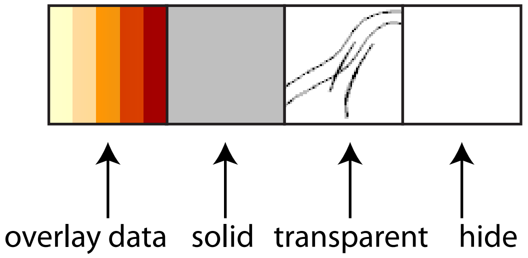

Technical Notes

This PDF file is preferably viewed in Adobe Reader® 9.3 or higher (http://www.adobe.com/downloads/)
Javascript must be enabled.

Open *Edit* ⇨ *Preferences* to ensure the following:

1) In *3D& Multimedia*, under *3D Tool Options*
   - for *Open Model Tree on 3D Activation* choose *Use Annotation's Settings*
   - for *Default Toolbar State* choose *Use Annotation's Settings*
   - disable *Show 3D Orientation Axis*
2) In *3D& Multimedia*, under *Auto-Degrade Options*
   - for *Optimalization Scheme for Low Framerate* select *None*
3) In *JavaScript*, under *JavaScript*
   - enable *Enable Acrobat JavaScript*

Cell cycle length in stage HH9 chicken embryo

views

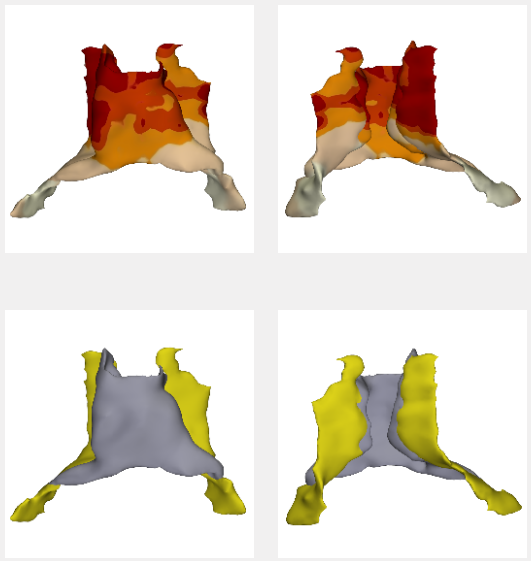

structures

myocardium

mesoderm

cell cycle length (hrs)

0 8 16 24 32 ∞

http://3d.hfrc.nl

Cell cycle length in stage HH12 chicken embryo

views

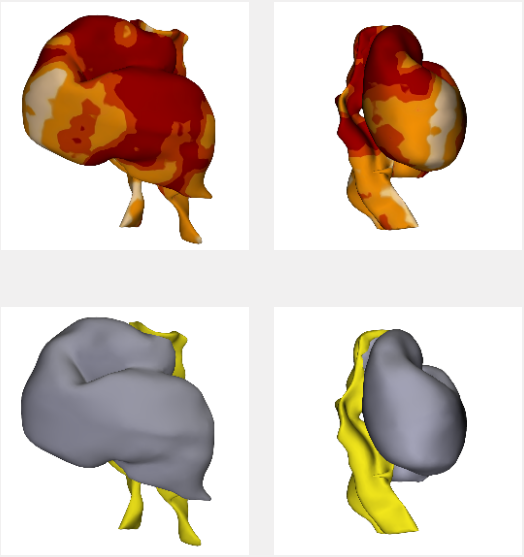

structures

myocardium

mesoderm

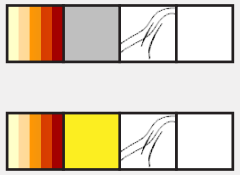

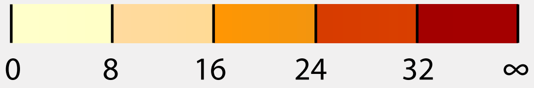

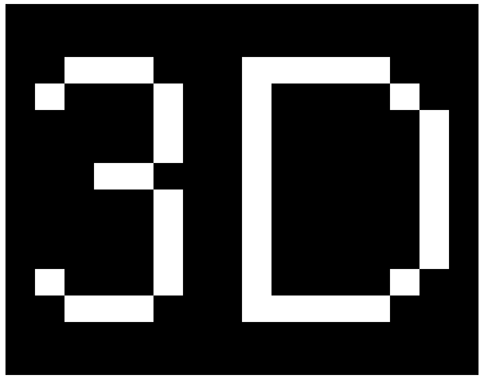

Cell cycle length in stage HH16 chicken embryo

views

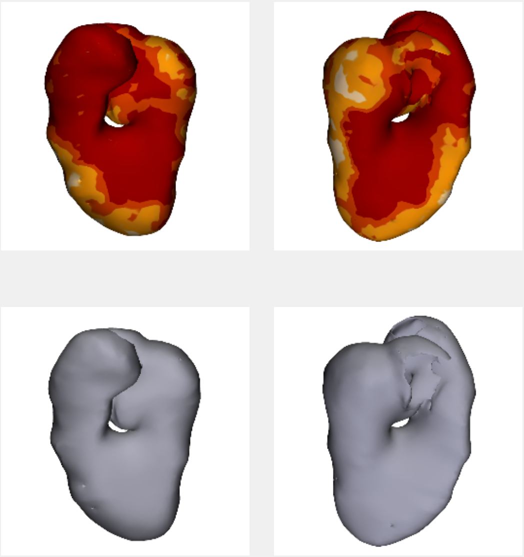

structures

myocardium

cell cycle length (hrs)

0 8 16 24 32 ∞

http://3d.hfrc.nl
