# Supplementary material for: Measurement and 3D-Visualization of Cell-Cycle Length Using Double Labelling with Two Thymidine Analogues Applied in Early Heart Development
Source: PLoS One. 2012 Oct 16;7(10):e47719. doi: 10.1371/journal.pone.0047719 (PMC3473012; doi:10.1371/journal.pone.0047719)
Supplement: Appendix S1 — Mathematics of labelling indices. This appendix describes the derivation of the equations used to calculate the cell cycle length and S-phase length from the labelling indices that can be observed after differential exposure of dividing cells to thymidine analogues. (PDF) [file pone.0047719.s003.pdf]

Supporting Information De Boer and Van den Berg et al. (2012). Appendix S1

Mathematics of labelling indices

When the dividing cells can be assumed to be in a random phase of the cell cycle, the cell cycle length is constant and the population does not increase in size during the exposure time, the fraction labelled cells (*F*) is determined by the exposure time and the unknown lengths of the S-phase and the cell cycle [1,2]. When two different exposure times are used, the unknown lengths can be derived.

Derivation of the equation for T_C

According to the equation given by Sanders and co-workers [1] the labelling fraction after CldU exposure is given by:

$$F_{Cl} = \frac{T_S + T_{Cl}}{T_C} \qquad [1]$$

which can be solved for T_S:

$$T_S = F_{Cl} T_C - T_{Cl} \qquad [2]$$

Similarly, the exposure to IdU results in the labelling fraction of:

$$F_I = \frac{T_S + T_I}{T_C} \qquad [3]$$

Substitution of Eq. [2] into Eq. [3] gives:

$$F_I = \frac{F_{Cl} T_G - T_{Cl} + T_I}{T_C} \qquad [4]$$

which can be solved for T_C:

$$T_C = \frac{T_I - T_{Cl}}{F_I - F_{Cl}} \qquad [5]$$

With Eq. [6] it is easy to see that an incorporation lag (T_L) has no effect on observed T_C, because such a lag affects both exposure times in the same way:

$$T_C = \frac{(T_I - T_L) - (T_{Cl} - T_I)}{F_I - F_{Cl}} = \frac{T_I - T_{Cl}}{F_I - F_{Cl}} \qquad [6]$$

The labelling fraction after exposure to CldU for a population with a growth fraction (F_D) is given by Equation 1a [1,2]. This equation is used to derive the equation for T_C, including the growth fraction (the equations are in the same order as above):

$$F_{Cl} = \frac{T_S + T_{Cl}}{T_C} \cdot F_D \qquad [1a]$$

which can be solved for $T_S.F_D$:

$$T_S \cdot F_D = F_{Cl} \cdot T_C - T_{Cl} \cdot F_D \qquad [2a]$$

The similar exposure to IdU results in the labelling fraction of:

$$F_I = \frac{(T_S + T_I) \cdot F_D}{T_C} = \frac{T_S \cdot F_D + T_I \cdot F_D}{T_C} \qquad [3a]$$

Substitution of Eq. [2a] into Eq. [3a] results in:

$$F_I = \frac{F_{Cl} \cdot T_G - T_{Cl} \cdot F_D + T_I \cdot F_D}{T_C} \qquad [4a]$$

which can be simplified to:

$$T_C = \frac{T_I - T_{Cl}}{F_I - F_{Cl}} \cdot F_D \qquad [5a]$$

Equation 5a shows that the observed T_C of a population, i.e. the population doubling time, is the cell cycle length of the dividing cells multiplied by the growth fraction of the population.

Derivation of the equation for Ts

The equation given by Sanders and co-workers [1] states that the labelling index after exposure to CldU is given by:

$$F_{Cl} = \frac{T_S + T_{Cl}}{T_C} \qquad [7]$$

which, solved for T_C reads like:

$$T_C = \frac{T_S + T_{Cl}}{F_{Cl}} \qquad [8]$$

Similarly the exposure to IdU results in the labelling fraction of:

$$F_I = \frac{T_S + T_I}{T_C} \qquad [9]$$

Substitution of Eq. [8] in Eq. [9] then gives

$$F_I = \frac{T_S + T_I}{\left(\frac{T_S + T_{Cl}}{F_{Cl}}\right)} \qquad [10]$$

This can be rearranged to give the following equation for T_S:

$$T_S = \frac{F_{Cl} \cdot T_I - F_I \cdot T_{Cl}}{(F_I - F_{Cl})} \qquad [11]$$

Equation 11 can be simplified to:

$$T_S = F_{Cl} \cdot T_C - T_{Cl} \qquad [11a]$$

as was derived in Figure 1.

When the growth fraction (F_D), which is the fraction of cells that is dividing is constant, T_S is derived as follows (the equations are in the same order as above):

$$F_{Cl} = \frac{(T_S + T_{Cl})}{T_C} \cdot F_D \qquad [7a]$$

$$T_C = \frac{(T_S + T_{Cl})}{F_{Cl}} \cdot F_D \qquad [8a]$$

$$F_I = \frac{(T_S + T_I) \cdot F_D}{T_C} \qquad [9a]$$

$$F_I = \frac{(T_S + T_I) \cdot F_D}{\left(\frac{T_S + T_{Cl}}{F_{Cl}}\right) \cdot F_D} \qquad [10a]$$

In Eq. [10a] it is clear that F_D disappears from the equation. Therefore, the growth fraction has no influence on the calculation of the S-phase length.

The bias due to the insertion a lag time (T_L) between injection and incorporation, on determined S-phase is equal to the incorporation lag. With the definition of T_S in Eq. 11 and a lag phase T_L, the real T_S can be defined as

$$T_S \operatorname{Re}al = \frac{F_{Cl} \cdot (T_I - T_L) - F_I \cdot (T_{Cl} - T_L)}{(F_I - F_{Cl})} \qquad [12]$$

which after re-arranged reads as:

$$T_S \operatorname{Re}al = \frac{F_{Cl} \cdot T_I - F_I \cdot T_{Cl}}{(F_I - F_{Cl})} + \frac{F_I \cdot T_L - F_{Cl} \cdot T_L}{(F_I - F_{Cl})} \qquad [13]$$

The first part on the right is the T_S that will be observed because the presence of a lag phase is unknown, the second part simplifies to T_L:

$$T_S \,\mathrm{Re}\,al = T_S Obs + T_L \quad [14]$$

Equation 14 shows that the observed S-phase length is too short when an incorporation lag is present. The length of this lag has to be added to obtain the real S-phase length.

The actual underestimation of the observed S-phase length would thus be equal to such an incorporation lag. The effect of this lag phase explains the discrepancy between our S-phase equation (11a) and those previously published [3,4]: by assuming a lag phase that is as long as the exposure time to the second label, these authors ignore the second exposure time and the S-phase length is thus overestimated by the length of this exposure time.

Division of labelled cells

When the exposure time to the first label (IdU) is longer than T_{G2} + T_M, cells that were labelled during T_S reach the end of T_M and will divide. In that case, the fraction of labelled cells at the moment of fixation that was defined as F_I, is also equal to the fraction of cells in S, G2 and M plus a fraction of cells that results from the cell division:

$$F_I = F_S + F_{G2} + F_M + F_{division} \quad [15]$$

or

$$F_{division} = F_I - \left(F_S + F_{G2} + F_M\right) \quad [16]$$

With

$$F_S + F_{G2} + F_M = \frac{T_S + T_{G2} + T_M}{T_C} \quad [17]$$

and Eq. 3 for F_I, $F_{division}$ (Eq. 16) can be re-written as

$$F_{division} = \frac{T_S + T_I}{T_C} - \frac{T_S + T_{G2} + T_M}{T_C} \quad [18]$$

which simplifies to

$$F_{division} = \frac{T_I - \left(T_{G2} + T_M\right)}{T_C} \quad [19]$$

Equation [19] shows that for exposure times (T_I) longer than the sum of T_{G2} and T_M, an extra group of dividing cells is counted and added to F_I. This will increase the

denominator in Eq. 5 and the observed T_C will thus be underestimated. Therefore, the exposure time should be kept shorter than the sum of T_{G2} and T_M.

References

1. Sanders EJ, Varedi M, French AS (1993) Cell proliferation in the gastrulating chick embryo: a study using BrdU incorporation and PCNA localization. Development 118: 389-399.
2. Nowakowski RS, Lewin SB, Miller MW (1989) Bromodeoxyuridine immunohistochemical determination of the lengths of the cell cycle and the DNA-synthetic phase for an anatomically defined population. J Neurocytol 18: 311-318.
3. Shibui S, Hoshino T, Vanderlaan M, Gray JW (1989) Double labeling with iodo- and bromodeoxyuridine for cell kinetics studies. J Histochem Cytochem 37: 1007-1011.
4. Martynoga B, Morrison H, Price DJ, Mason JO (2005) Foxg1 is required for specification of ventral telencephalon and region-specific regulation of dorsal telencephalic precursor proliferation and apoptosis. Dev Biol 283: 113-127.
